# Management of nipple adenomas during pregnancy: a case report

**DOI:** 10.1186/s13006-023-00554-4

**Published:** 2023-03-21

**Authors:** Francesca Combi, Enza Palma, Giulia Montorsi, Anna Gambini, Silvia Segattini, Simona Papi, Alessia Andreotti, Giovanni Tazzioli

**Affiliations:** 1 International PhD School in Clinical and Experimental Medicine (CEM), University of Modena and Reggio Emilia, Modena, Italy; 2 Division of Breast Surgical Oncology, Department of Medical and Surgical, Maternal-Infantile and Adult Sciences, University Hospital of Modena, Modena, Italy; 3 General Surgery Residency Program, University of Modena and Reggio Emilia, Modena, Italy

**Keywords:** Nipple adenoma, Breastfeeding, Lactation, Pregnancy, Benign breast lesion, Nipple preservation

## Abstract

**Background:**

Nipple adenoma is a very uncommon, benign neoplasm that involves the nipple. A palpable mass of the nipple associated with nipple discharge and erosion or ulceration is the common clinical presentation. Generally, complete surgical excision of the nipple is the main treatment, alternative therapeutic methods such as Mohs micrographic surgery, nipple splitting enucleation, and cryotherapy can be considered. Disorders of the breast in young women are generally benign. Even if the management during pregnancy is usually conservative and surgical excision is reserved for very strong malignancy suspicion, benign lesions can cause the impossibility to breastfeed after giving birth when involving the nipple.

**Case presentation:**

We present the case of a 28-year-old female, who was referred to the Breast Unit of the University Hospital of Modena (Italy) in May 2020 with a 12-months history of enlargement of the left nipple with associated erythema, serohemorrhagic discharge, and pain in the left nipple region. The diagnostic assessment came out in favor of a nipple adenoma. After surgical treatment was recommended, the patient got pregnant. Taking into account the major risks of surgery during pregnancy, a multidisciplinary discussion was conducted, to consider whether to proceed with surgery or postpone it after pregnancy. Because of the volume and the position of the adenoma, the indication for surgical excision was confirmed, to allow regular lactation and breastfeeding immediately after giving birth and to avoid potential obstructive complications. Surgical excision of nipple adenoma without complete resection of the nipple was performed after her first trimester of pregnancy under local anesthesia. A histopathological examination confirmed the diagnosis. No recurrence occurred after 12 months. The patient gave birth, had no deficit in lactation, and successfully breastfed.

**Conclusions:**

Therefore, we consider that nipple adenoma enucleation might be a safe treatment even during pregnancy. Moreover, conservative local treatment of nipple adenomas can preserve the nipple aesthetically and functionally, thus allowing regular lactation and breastfeeding in young women.

**Supplementary Information:**

The online version contains supplementary material available at 10.1186/s13006-023-00554-4.

## Background

Nipple adenoma is a rare benign breast proliferative process of lactiferous ducts that usually affects middle-aged women. A palpable mass of the nipple associated with nipple discharge and erosion or ulceration is the common clinical presentation [1]. Nipple adenoma can be misdiagnosed clinically as a malignant nipple lesion (like mammary Paget’s disease [MPD]), histologically as intraductal carcinoma [2, 3]. The key to obtaining early diagnosis and assessment for concomitant malignancy is represented by multimodality radiological evaluation employing mammography, ultrasonography, or breast MRI. Dermoscopy in combination with histopathologic studies can be useful for accurate diagnosis [4]. Histologically, nipple adenomas show an irregular ductal proliferation within nipple stroma without significant cellular atypia. Treatment of choice consists of complete surgical excision to prevent local recurrence, other treatments include Mohs micrographic surgery, nipple splitting enucleation technique, cryotherapy, or radiofrequency. Local recurrence is not infrequent, especially in the case of incomplete excision [5, 6]. Although the coexistence of nipple adenoma and ipsilateral or contralateral breast cancer has been reported, this association is difficult to establish [7].

Disorders of the breast in young women are generally benign. Although rare, nipple adenoma and other benign breast disorders (BBD) can also be seen during pregnancy and lactation. Management of BBD during pregnancy is usually conservative and surgical excision is only mandatory in case of rapid enlarging or discordance in the triple diagnostic assessment [8].

We report herein a case of adenoma of the nipple in a young woman, surgically managed during pregnancy by curative tumor resection without excision of the nipple. At last, we summarized the indications and management of nipple adenomas in pregnant women.

## Case presentation

We present the case of a 28-year-old female, who was referred to the Breast Unit of the University Hospital of Modena (Italy) in May 2020 with a 12-months history of enlargement of the left nipple with associated erythema, serohemorrhagic discharge, and pain in the left nipple region. Her past medical history was unremarkable. Positive family history of early-stage breast cancer was found in her mother, at the age of forty-eight. The patient had noticed the mass about 12 months before her consultation and during that interval the lesion had slowly increased in size, causing mild symptoms of pressure and discomfort.

**Fig. 1:**
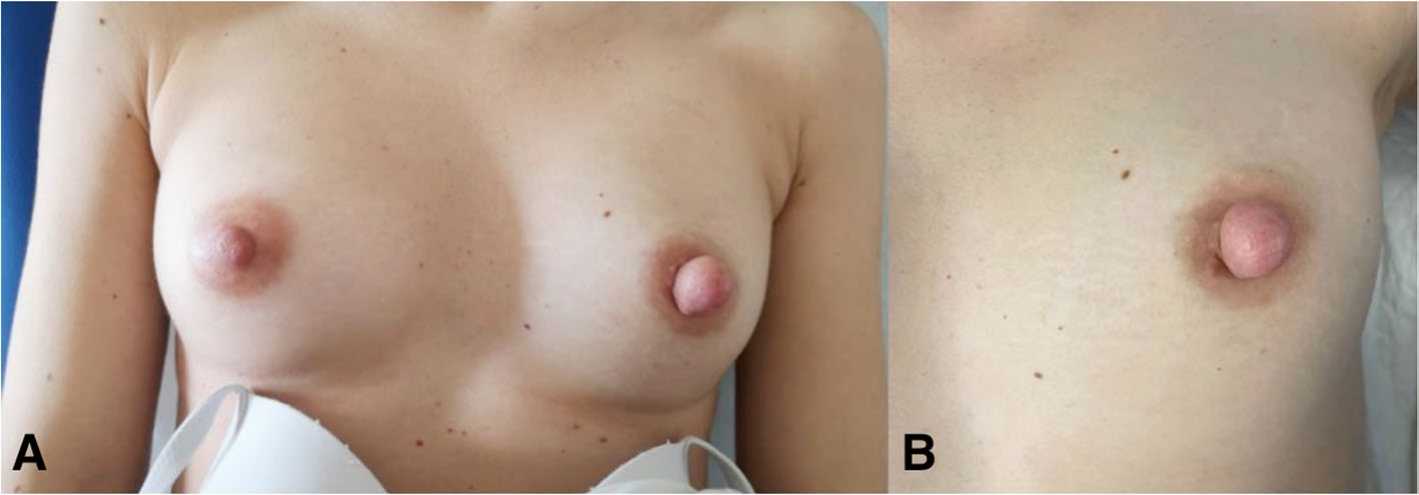
Clinical presentation at the onset. **a** shows both breasts at the onset; **b** shows the left breast with nipple adenoma

On physical examination, the left nipple was enlarged and twice the size of the contralateral, a non-tender well circumscribed movable mass of approximately 10 mm was palpable, as shown in Fig. 1. Serous fluid discharge from a single orifice of the left nipple appeared with squeezing. Neither intraparenchymal breast masses nor axillary lymph nodes were palpable on clinical breast examination. Bilateral breast ultrasonography revealed an oval, well-demarcated hypoechoic lesion in the left nipple measuring 10 × 7 mm. No other abnormal ultrasonographic findings within the region behind the left nipple-areola complex or elsewhere within the left or right breast were detected.

On the fine-needle aspiration cytology (FNAC) examination serous-haematic material with low to moderate cellularity within epithelial hyperplastic cellular nests and myoepithelial units was reported. This pattern was suggestive of a benign adenomatous process (C3 cytological classification) with no signs of atypia or malignancy [9]. Fine-needle aspiration biopsy (FNAB) was not performed since it was considered technically unfeasible, due to the position and projection of the lesion, out the top of the nipple. Furthermore, the surgical team recommended excision, both to reach a certain histological diagnosis and for the dimension of the mass, which was rapidly growing and distorting the nipple profile. Full nipple excision was never offered as the first surgical option, because of the young age of the patient and the will to preserve both functional and aesthetic aspects of the nipple-areola complex.

**Fig. 2:**
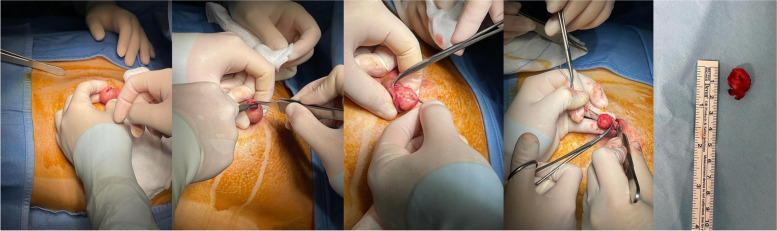
Surgical enucleation of left nipple adenoma

**Fig. 3:**
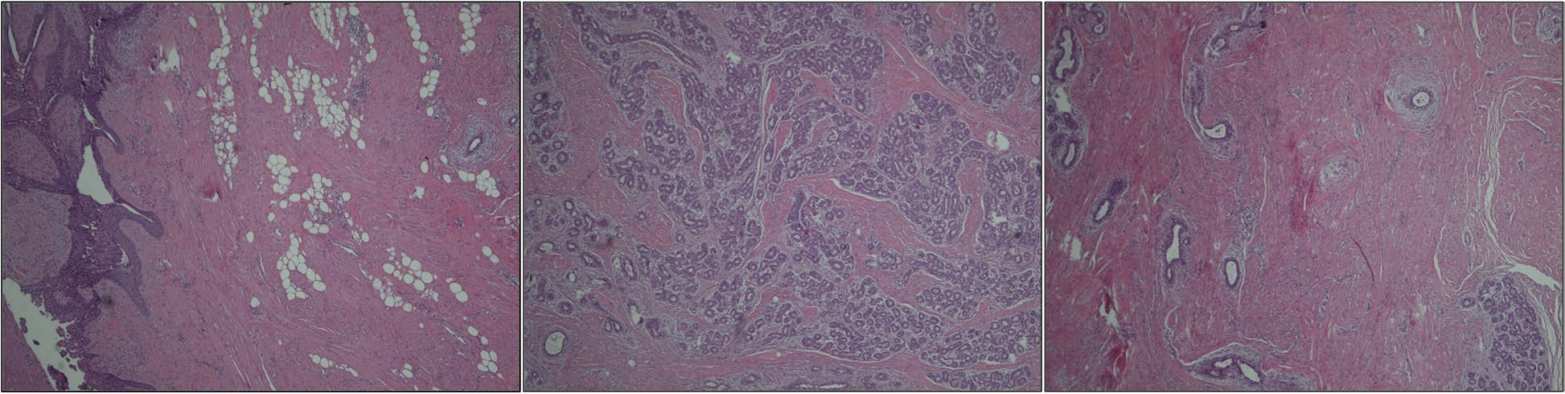
Histology of the lesion with hematoxylin-eosin staining

The surgical indication was to perform the excision of the mass under local anesthesia, with preservation of the nipple. During the waiting period for the surgical procedure, the patient became pregnant. She has been referred once again to our Breast Unit for scheduled mammary ultrasonographic control in her sixth week of pregnancy. Taking into account the major risks of surgery during pregnancy, a multidisciplinary discussion was conducted, to consider whether to proceed with surgery or postpone it after pregnancy. Because of the volume and the position of the adenoma, the indication for surgical excision was confirmed, to allow regular lactation and breastfeeding immediately after giving birth and to avoid potential obstructive complications such as galactocele, breast abscess, or mastitis. The end of the first trimester was established as the ideal time for the procedure. The patient underwent surgery in her fifteenth week of pregnancy. The surgical excision of nipple adenoma was performed under local anesthesia with mepivacaine by an experienced breast surgeon. A nipple splitting enucleation technique was performed through a 1 cm trans-nipple longitudinal incision made down through the long axis of the nipple profile, to expose and radically enucleate the mass, as shown in Fig. 2 and Additional File Surgical Videos (Additional file 1 and Additional file 2). Nipple reconstruction was obtained with non-absorbable interrupted sutures to restore the original shape. Although the tumor projection was out the top of the nipple, curative resection without total excision of the nipple was possible, and the left nipple was cosmetically and functionally preserved. Histopathologic evaluation by a breast expert pathologist revealed on gross examination a circumscribed gray nodule measuring 20 × 15 × 10 mm; the cut surface was grayish and friable. The microscopical evaluation showed a fibroepithelial benign lesion with a stromal component enriched with myoepithelial cells of the outer layer highlighted using antibodies against calponin and desmin. Lactiferous ducts appeared coated by typical ductal hyperplasia. The overlying epidermis was not directly involved by the adenoma and epidermal ulceration was not identified. Therefore, these findings were suggestive of nipple adenoma. The histology of the lesion with hematoxylin-eosin staining is showed in Fig. 3.

**Fig. 4:**
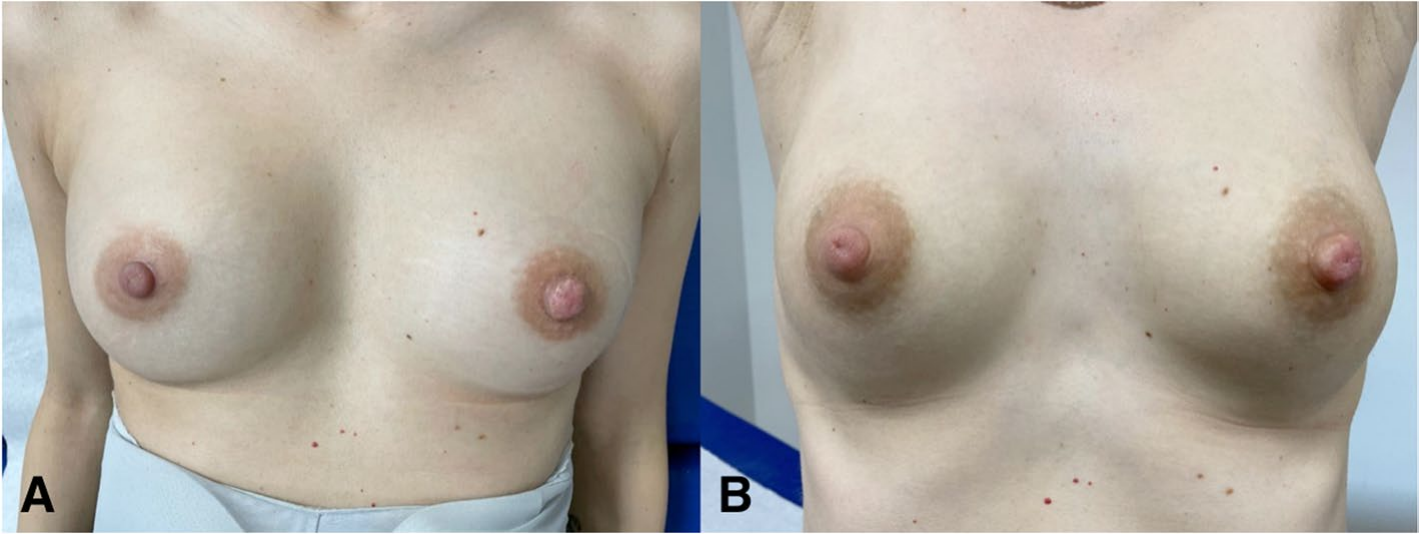
Post-surgical results. **a** shows both breasts two months post-surgery; **b** shows both breasts nine months post-surgery

The postoperative course was uneventful. Outpatient monitoring showed a correct wound-healing process and a progressive reduction of the left nipple size. Figure 4 shows post-operative results 2 months and 9 months after surgery. For regularly scheduled follow-ups we planned an annual clinical breast examination. The patient gave birth at the end of May 2021 and had a regular history of lactation and breastfeeding, which is still ongoing at present, 9 months after surgery. Moreover, the patient remains satisfied with the final aesthetic result.

## Discussion

Nipple Adenoma (NA), also known as erosive adenomatosis or florid papillomatosis of the nipple, is a benign proliferation of the lactiferous ducts of the nipple [7, 10]. It was first described as a distinct clinicopathological entity in 1955 by Jones et al. [11]. Nipple adenomas typically present in women in the fourth and fifth decades of life, exceptionally they have also been reported in men or children, and they generally occur unilaterally. The initial clinical presentation is a hard-elastic nodule that distorts the nipple profile, usually causing swelling, inflammation, erythema, or erosion with serous or haematic discharge, possibly associated with pain in the nipple-areolar region [1]. Our patient showed a single tender palpable tumor, increasing the left nipple dimension. The World Health Organization (WHO) classification of breast tumors established in 2012 [10], defined nipple adenomas as a compact proliferation of small tubules lined by layers of epithelial and myoepithelial cells, with or without proliferation of the epithelial component, around the collecting ducts of the nipple [12]. Although nipple adenoma represents a rare benign neoplasm, the main issue is the differential diagnosis with nipple Paget’s disease, DCIS of low-grade, syringomatous adenoma, and subareolar solitary central papilloma [2, 3].

This rare entity, which accounts for just 1–1.7% of benign breast lesions [13], can include various histological patterns. The main histological feature is the ductal proliferation of glandlike structures within the stroma of the nipple, with well-circumscribed margins without encapsulation [7, 12–15]. Confirmation of the presence of at least two distinct layers of myoepithelial cells in neoplastic ducts seems to be the most important finding for the differential diagnosis of ductal carcinoma. Immunohistochemical staining using CD10, p63, alfa-smooth muscle actin, calponin, or desmin can be useful for myoepithelial cell detection in neoplastic ducts. An adequate histological and immunophenotypic analysis is recommended for discriminating the pseudo-invasive pattern from breast cancer precursors and aggressive carcinoma [12, 16, 17].

Despite its benign behavior, the expanding growth pattern and frequent local recurrence could harm a patients’ quality of life, especially for women of childbearing age who need to maintain intact the function of the nipples. Moreover, the presence of an abnormal mass of the nipple could affect the baby’s ability to latch and suck during breastfeeding.

Association with malignant breast carcinoma has been previously described; with regards to the probability of a tumor developing from nipple adenoma, no reliable data are available so far [18, 19].

The optimal management of benign nipple lesions during pregnancy is controversial and scarce evidence has been produced [8]. To our knowledge, this is the first case report to be published regarding the management of nipple adenomas in pregnant women. On one hand, non-urgent surgical procedures are generally avoided during pregnancy, to minimize the risks of anesthesia and surgical complications that can negatively affect the mother and the fetus. On the other hand, nipple lesions can represent a major impairment in the physiological process of lactation and breastfeeding, if not promptly treated. Even during pregnancy and breastfeeding, it is mandatory for any palpable lesion or visual change of the breast, to be evaluated with a thorough and complete examination (the gold standard remains the triple assessment, i.e., clinical, radiological and histological sampling of any lesion) to reach the certainty of the benign nature. Once the triple assessment is complete and the histological diagnosis is ascertained, management of benign disorders of the breast during pregnancy is usually conservative. Surgery is mandatory only in case of rapid enlarging or symptomatic lesions or to reduce the risk of future breastfeeding impairment [8]. Although the total excision of the nipple-areola complex with associated underlying wedge resection of the breast parenchyma or complete resection of the nipple is reported in the literature, these procedures could be considered overly aggressive for benign lesions [20, 21]. Since nipple adenoma represents a completely benign lesion, we recommend considering local surgical excision as the appropriate first-choice treatment. In this case, we performed a curative resection without nipple complete excision. Breast surgical techniques that preserve the column of subareolar parenchyma appear to have a greater potential for successful breastfeeding, in our case surgical enucleation of nipple adenoma with preservation of some of the lactiferous ducts has granted successful lactation even on the affected breast.

Regularly scheduled follow-up is recommended in these patients, regardless of the therapeutic methods, since nipple adenoma’s risk of recurrence or progressing into a malignancy can not be fully excluded.

## Conclusions

In conclusion, we reported a rare case of nipple adenoma and local excision in a pregnant young female. Traditional complete removal of the nipple may result in overtreatment, make breastfeeding impossible and an unsatisfactory aesthetic outcome. Conservative local treatment can be considered after the end of the first trimester of pregnancy and can allow the functional preservation of the nipple in young women.

## Supplementary Information

**Additional file 1.** Surgical Procedure – part1. Enucleation of left nipple adenoma with preservation of lactiferous ducts.

**Additional file 2.** Surgical Procedure – part 2. Complete excision of left nipple adenoma.

## Data Availability

The data used to support the findings of this study are included in the article and the supplementary information files.

## Abbreviations

BBD: Benign breast disorders
DCIS: Ductal carcinoma in situ
FNAB: Fine-needle aspiration biopsy
FNAC: Fine-needle aspiration cytology
MPD: Mammary Paget’s Disease
MRI: Magnetic resonance imaging
NA: Nipple adenoma
